# Epstein-Barr Virus and the Origin of Myalgic Encephalomyelitis or Chronic Fatigue Syndrome

**DOI:** 10.3389/fimmu.2021.656797

**Published:** 2021-11-15

**Authors:** Manuel Ruiz-Pablos, Bruno Paiva, Rosario Montero-Mateo, Nicolas Garcia, Aintzane Zabaleta

**Affiliations:** ^1^ Faculty of Medicine of the European University of Madrid, Madrid, Spain; ^2^ Clinica Universidad de Navarra, Centro de Investigación Medica Aplicada (CIMA), IdiSNA, Instituto de Investigación Sanitaria de Navarra, Pamplona, Spain; ^3^ Department of Pediatrics, Hospital Clínico San Carlos, Madrid, Spain

**Keywords:** chronic fatigue syndrome, myalgic encephalomyelitis, EBV EBNA-1, HLA-II alleles, cancer, CD4+ CTL, autoimmunity, immunotherapy

## Abstract

Myalgic encephalomyelitis or chronic fatigue syndrome (ME/CFS) affects approximately 1% of the general population. It is a chronic, disabling, multi-system disease for which there is no effective treatment. This is probably related to the limited knowledge about its origin. Here, we summarized the current knowledge about the pathogenesis of ME/CFS and revisit the immunopathobiology of Epstein-Barr virus (EBV) infection. Given the similarities between EBV-associated autoimmune diseases and cancer in terms of poor T cell surveillance of cells with EBV latency, expanded EBV-infected cells in peripheral blood and increased antibodies against EBV, we hypothesize that there could be a common etiology generated by cells with EBV latency that escape immune surveillance. Albeit inconclusive, multiple studies in patients with ME/CFS have suggested an altered cellular immunity and augmented Th2 response that could result from mechanisms of evasion to some pathogens such as EBV, which has been identified as a risk factor in a subset of ME/CFS patients. Namely, cells with latency may evade the immune system in individuals with genetic predisposition to develop ME/CFS and in consequence, there could be poor CD4 T cell immunity to mitogens and other specific antigens, as it has been described in some individuals. Ultimately, we hypothesize that within ME/CFS there is a subgroup of patients with DRB1 and DQB1 alleles that could confer greater susceptibility to EBV, where immune evasion mechanisms generated by cells with latency induce immunodeficiency. Accordingly, we propose new endeavors to investigate if anti-EBV therapies could be effective in selected ME/CFS patients.

## Introduction

Myalgic encephalomyelitis or chronic fatigue syndrome (ME/CFS) is a life-limiting, multi-system disease for which there is no effective treatment (1). It is characterized by unexplained disabling fatigue and a combination of unspecific symptoms that last for at least 6 months (2, 3). At least nine disease definitions have been developed. Its prevalence ranges in between 0.1% and 2.5% of the general population (4), depending on the diagnostic criteria being applied (**Supplemental Table 1**). The annual incidence of cases with ME/CFS in the United Kingdom is of 14.8 per 100,000 people (5).

### Pathogenesis of ME/CFS

The cause of this syndrome is unknown. However, there is a growing body of evidence supporting the role of dysfunction in immune, neuro-endocrine, and autonomic systems, and several biologically based theories are currently being investigated. Hormonal alterations have been identified in some individuals with ME/CFS, whereby hypocortisolism might produce fatigue-like symptoms (1, 6–8). Lipid and energy metabolism dysfunction are also thought to contribute to the etiology of ME/CFS. Genetic predisposition or common environmental exposure to infectious or toxic agents, may be associated with some family histories with high prevalence of ME/CFS (1, 9–14).

Infectious triggering a chronic inflammatory response has long been a hypothesized risk factor for the development of ME/CFS due to the large number of individuals with a history of infection prior to the onset of symptoms. Clinical manifestations of the disease such as chronic fatigue and flu-like symptoms, may be explained by the presence immunological alterations leading to reduced cytotoxic activity and altered metabolism of natural killer (NK) cells and T lymphocytes, reduced T cell responses to mitogens and other specific antigens, or the presence of chronic low-grade systemic inflammation with increased levels of proinflammatory cytokines and oxidative stress (1, 2, 15–18).

Another hypothesis is autoimmunity due to the presence of autoantibodies against nuclear, membrane and neurotransmitter receptor structures in some patients (1, 19). This hypothesis has prompted several research groups to seek for an association between the expression of certain HLA-II alleles and the development of ME/CFS. Accordingly, HLA-DQA1*01, HLA-DQB1*06 (20), DQB1*0303 (21), HLA-DQ3, HALA-DR4 and HLA-DR5 (22) have been associated with increased risk of ME/CFS, but the robustness of the data supporting this association is limited (20, 23). Thus, the possibility that some individuals with genetic predisposition may develop ME/CFS after stimulus (e.g., infection) and subsequent autoimmunity, remains to be demonstrated (12, 20, 22). In such cases, the diagnostic criteria could take into account the pathogen involved in the disease onset, which could potentially improve patient’ stratification and management.

Interestingly, ME/CFS was first described in reference to a post Epstein-Barr fatigue. Infection with viruses such as Epstein-Barr (EBV), but also with human herpesvirus (HHV) -6, cytomegalovirus (CMV), human parvovirus B19 and enteroviruses (24–30), as well as bacterial and parasite infections (31), have been suggested as risk factors. However, infection prior to its onset is not true of all ME/CFS patients and its etiological significance remains uncertain.

Here, we revisited the immunopathobiology of EBV infection before summarizing contradictory data about a possible association between chronic EBV infection and ME/CFS in some patients. Consistent with this hypothesis, we finalized this mini review describing possible therapeutic options against EBV.

## Immunopathobiology of the Epstein-Barr Virus

**Figure 1:**
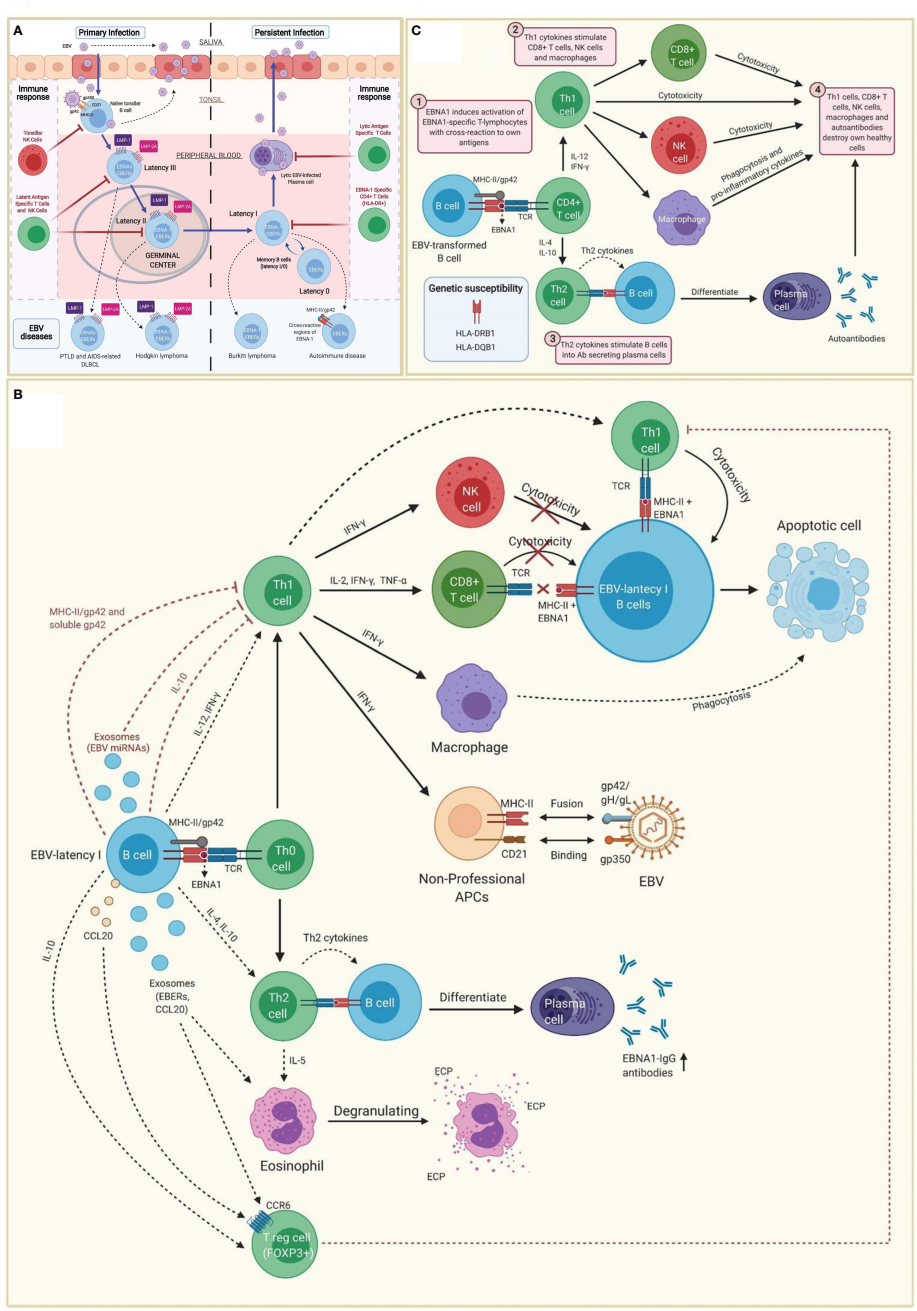
Immunopathobiology of Epstein-Barr virus (EBV) infection. **(A)** EBV is transmitted to the host through saliva from a carrier individual, and infects pharyngeal epithelial cells followed by naïve tonsillar B cells through interactions between gp350 and gp42 glycoproteins of the viral envelope with CD21 and MHC class II molecules (MHC-II), respectively. Lytic infection produces new viral particles that infect more epithelial cells. Subsequently, these EBV-infected B cells enter into a latency phase in the periphery, where they express a specific set of viral genes, including LMP1, LMP2A, EBNAs and EBER (latency III). These latent III B cells progress through the germinal center reaction into latency II and emerge as memory B cells with I/0 latencies that establish a lifelong latent infection. The immune response of the healthy host is sufficient to maintain control of the EBV infection. NK cells in tonsil produce high levels of IFN-γ that withhold the transformation of B cells by EBV during earlier stages of the infection. Both, type III and type II latent B cells are controlled by NK and T cells specific to latent proteins. By contrast, memory B cells with type I latency are only controlled by activated EBNA-1 specific CD4 T cells. EBV-infected plasma cells can periodically enter in lytic phase, but are controlled by CD4 and CD8 T cells with specificity for EBV lytic proteins. The programs of the viral latent cycle are expressed in various EBV-associated diseases. Latency I is found in Burkitt lymphoma, latency II in Hodgkin lymphoma and latency III in post-transplantation lymphoproliferative disease (PTLD) and AIDS-associated diffuse large B-cell lymphoma (DLBCL). EBV-latency I B cells escaping the surveillance of EBNA-1-specific CD4 T cells could lead to autoimmunity by presenting EBNA-1 in MHC-II/gp42, which may cause cross-reaction with own antigens. **(B)** EBNA1 is presented to CD4 T cells on MHC class II molecules in EBV-infected B cells. Both, MHC-II bound gp42 and soluble gp42 facilitate immune evasion by preventing activation and recognition of T cell receptors (TCR) in CD4 T cells. In addition, some EBV miRNAs could directly reduce CD4 T cell cytotoxicity through the intercellular exosomal pathway or inhibit MHC class II-mediated antigen processing and presentation in the host cell. By contrast, activation of Th1 CD4 T cells, would favor co-stimulation of CD8 T cells, NK cells and macrophages. Increased levels of IFN-γ in response to EBV infection can induce expression of MHC class II molecules in other cells such as epithelial cells, endothelial cells, pancreatic beta cells, fibroblasts, keratinocytes and glial cells, allowing them to act as non-professional antigen-presenting cells that can become infected by EBV through gp42/MHC-II interaction. If these cells also express low levels of CD21 (thymocytes, a subset of peripheral T lymphocytes, follicular dendritic cells, astrocytes and some epithelial cells), they may further facilitate EBV entry by interacting gp350 with CD21. IFN-γ released by NK cells may withhold the transformation of B cells by EBV during the early stages of infection, but it fails to inhibit the proliferation of fully transformed EBV-infected B cells (latency). CD8 T cells do not recognize EBNA1 in EBV-transformed cells, since it is presented in MHC class II molecules. Only EBNA1-specific CD4 T cells have cytotoxic activity against EBNA1-expressing B cells, causing them to enter apoptosis and become phagocyted by macrophages. However, EBV transformed B cells release IL-10, TGF-β, CCL20 and exosomes (containing EBERs and CCL20), which attract T regulatory (Treg) cells to the site of infection inhibiting antigen-stimulated CD4 effector T cells. IL-10 released by EBV transformed B cells during EBNA1 presentation, favors a Th2 (over Th1) immune response that also inhibits CD8 T cells and NK cells. These EBNA1 specific Th2 cells further induce antibody secretion by plasma cells. EBERs released on exosomes can activate other cells such as eosinophils that degranulate and release the cationic eosinophilic protein (ECP). **(C)** EBNA1 is one of the main candidates in the generation of autoantibodies and EBNA1 specific self-reactive cytotoxic Th1 cells in genetically predisposed individuals. EBNA-1 is subjected to citrullination and presented in MHC class II molecules after macroautophagy in EBV-infected B cells. As gp42 binds peripherally to the β1 domain of the β chain of HLA-DR -DQ, it may confer greater susceptibility or resistance to this interaction, depending on the host DRB1* and DQB1* allele. Post-translational modifications, such as citrullination, may form neoantigens that can generate autoimmunity when recognized by CD4 T cells. If polarized into a Th2 phenotype, CD4 T cells will stimulate B cell differentiation into plasma cells that could secrete autoantibodies. Antigen-specific CD4 T cells with a Th1 cytokine pattern may have cytotoxic activity, apart from co-stimulating CD8 T cells, NK cells and macrophages. Together with autoantibodies produced by plasma cells, all these cell types would participate in the destruction of healthy cells and the development of autoimmunity.

EBV belongs to the family of γ-herpesvirus (32). Amongst other cell types, it infects B cells of adjacent lymphoid tissues before establishing a lifelong latent infection in memory B cells (I/0 latencies). Indeed, the virus maintains a latent state as an episome without expressing viral genes, allowing B cells with latency 0 and some B cells with latency I to escape immune surveillance (**Figure 1A**) (33–36).

Efficient B cell infection by EBV requires up to 5 envelope glycoproteins, whereby gp42 ultimately promotes entry of the virus into B cells through the interaction with host MHC class II molecules (37, 38). Gp42 binds to β1 domain of the β chain of HLA-DR -DQ, or –DP and blocks TCR-HLA-DR interactions impairing antigen presentation (37–39). Hence, gp42-expressing B cells show reduced ability to activate CD4 T cells (37). Furthermore, gp42 can be presented as a membrane protein bound to MHC-II molecules or in a soluble form (s-gp42). Both have been detected during the lytic phase in Burkitt lymphoma (latency I), suggesting that soluble gp42 is generated during EBV lytic infection and inhibits the presentation of HLA-II restricted antigens to T cells (40). Noteworthy, EBNA-1 specific cytotoxic CD4 T cells are decreased in patients with post-transplant lymphoproliferative disorders (41), in some pediatric forms of Burkitt lymphoma (42, 43), in EBV-positive lymphoma (44), in lymphomas associated with HIV infection (45), and in lymphoma infiltrating the central nervous system (45). By contrast, the immune response in healthy individuals is sufficient to control EBV infection (35, 46).

### Genetic Predisposition to EBV Infection

The diversity of human leukocyte antigen (HLA) molecules results from selective pressure during co-evolution with pathogens (47, 48). A characteristic of HLA diversity is the long-term persistence of allelic lineages, which causes trans-species polymorphisms to be shared among closely related species (48). In humans, there are 13 allelic lineages of DRB1 (48) and, according to the phylogenetic relationship between the different DRB genes of primates (hominoids, New World and Old World monkeys) described by Bontrop et al, the DRB1*04, *03 and *02 lineages are the oldest, with the DRB1*04 lineage being the most ancestral (49). Since EBV is the only human-adapted member of the genus Lymphocryptovirus, transferred to a hominid ancestor (50), it could be hypothesized that immune evasion mechanisms of the EBV have more effectively evolved among older allelic lineages of DRB1. Such an hypothesis could help explaining why individuals with haplotypes DR2-DQ6, DR3-DQ2 or DR4-DQ8 are less resistant to EBV infection and are at greater risk of developing EBV-related disorders (**Supplemental Table 2**) (34).

Other genetic predisposition factors to consider are glutamic acid 46 (E46) and arginine 72 (R72) from HLA class II molecules (51). R72 is fully preserved in the HLA-DR, HLA-DQ and -DP sequences (52); by contrast, E46 is conserved in all HLA-DR,-DP alleles and only in a small subset of HLA-DQ alleles β * 02 (β * 0201, β * 0202 and β * 0203) (53). This suggests that individuals with HLA-DQ alleles β * 02 may have higher susceptibility to infection in tissues with cells expressing only HLA-DQ, as well as a higher rate of EBV infection in those cells expressing different HLA class II isotypes along with HLA-DQ β * 02 (53).

### EBV-Associated Diseases

EBV is present in more than 90% of the population and the decreased ability of the immune system to control/eliminate EBV infection may be responsible for causing EBV-associated diseases (35, 54) such as rheumatoid arthritis (34, 55, 56), systemic lupus erythematosus (34, 57, 58), Sjögren’s syndrome (34), multiple sclerosis (59–61), myasthenia gravis (62), diabetes mellitus type 1 (53), fulminant type diabetes (63), celiac disease (64), autoimmune thyroiditis (65, 66), Hodgkin and non-Hodgkin lymphoma (35, 67) (**Supplemental Table 2**).

Tissue infiltrating B cells with EBV latency in a genetically predisposed individual, would trigger the activation of virus-specific IFN-γ producing Th1 CD4 T cells. High IFN-γ levels would induce the upregulation of MHC class II molecules (**Figure 1B**) on the surface of different cell types (e.g., epithelial cells, endothelial cells, pancreatic beta cells, fibroblasts, keratinocytes, glial cells, etc.), allowing them to function like non-professional antigen-presenting cells. Likewise, MHC class II upregulation would improve the infection capacity of EBV through the interaction with gp42 (68, 69). Co-expression of MHC-II molecules and CD21 (e.g., thymocytes, a subset of peripheral T lymphocytes, follicular dendritic cells, astrocytes and some epithelial cells) would further increase the risk of EBV infection through the interaction between gp350 and CD21 (69). These mechanisms have been hypothesized in patients with autoimmune thyroiditis with EBV transformed B cell infiltrate in the thyroid tissue (65, 66). The same principle could potentially apply to the intestinal mucosa in celiac disease (70), to the pancreatic islets in type 1 diabetes (53), to the central nervous system and multiple sclerosis (60, 71), to the exocrine glands and the Sjögren’s syndrome (34, 72), to the thymus and myasthenia gravis (62), as well as to the synovial joints and rheumatoid arthritis (38, 73, 74). Can the same principle be applied to a subgroup of patients with ME/CFS?

## A Hypothetical Association Between EBV and ME/CFS

As mentioned above, EBV infection has been identified as a risk factor in a subgroup of ME/CFS patients (3, 28, 75). There are studies showing a statistically significant elevation of anti-EBV-dUTPase antibodies (75, 76), a defective EBV-specific B and T cell response (77), a high rate of active EBV infection (28), serologic evidence of EBV reactivation with elevated IgM antibodies against late VCA antigen (78–80), and a positive up-regulation of EBV-induced gene 2 (EBI2) mRNA in peripheral blood mononuclear cells (PBMC) (3) from a subgroup of patients with ME/CFS. However, serological observations related to EBV were not always confirmed and, accordingly, the association between EBV infection and ME/CFS is not established (81–85). Furthermore, the presence of an active EBV infection in a subgroup of ME/CFS patients has been actively debated, because most studies revealed no increase in EBV viral load in ME/CFS patients. If a third state of virus, defined as abortive/lytic/leaky replication (86) could explain the presence of certain EBV proteins (BRRF1 and BLLF3) with the potential capacity to contribute to the symptomatology of ME/CFS (82, 87), is an hypothesis that remains unconfirmed.

Multiple studies in patients with ME/CFS have demonstrated decreased cytotoxic activity of NK cells, increased IL-10 levels and augmented Th2 response. Also an expansion of Tregs and impaired T cell response to mitogens and other specific antigens (1, 2, 12, 16, 88–91). Based on the mechanisms of immune-escape developed by latency I (EBNA-1) cells, established after primary EBV infection, a cause-effect association between EBV infection and the disease onset in a subgroup of ME/CFS patients, could be hypothesized. Accordingly, IL-10 released by both EBV-transformed B cells and Tregs would favor a Th2 type immune response (43), and gp42-mediated disruption of TCR-MHC-II interaction would further decrease CD4 T cell activation, leading to poor CD4 T cell immunity to mitogens and other specific antigens, which have been described in some patients with ME/CFS (2). All these immune evasion mechanisms triggered by latent cells induce an immunodeficiency that allows EBV-transformed B cells, especially EBV latent I cells, to escape from immune surveillance. This could potentially help explaining the reduced EBNA-1-specific CD4 T-cell response, increased EBV latent cells (77), and increased EBV abortive lytic replication (EBV dUTPase in exosomes) (75), observed in a subgroup of patients with ME/CFS. If the infiltration and proliferation of EBV-transformed B cells in the intestinal mucosa is related to the chronic inflammation that is observed in some ME/CFS patients (15, 92–94), remains unknown. However, the results described above and the hypotheses we generated accordingly, must be interpreted with caution because there are numerous other studies that failed to reproduce the findings of decreased cytotoxic activity of NK cells, increased IL-10 levels and augmented Th2 response (83, 89, 90, 95–97). These discrepancies may be due to disease heterogeneity, different degrees of ME/CFS severity and duration, and different analytical methods (15, 28, 89, 97–99).

If the hypotheses described above were to be true, the subgroup of ME/CFS patients with EBV infection could have increased risk of developing autoimmune diseases (19, 100, 101), latent viral reactivations (e.g., herpesviruses, Parvovirus B19) and cancer (83, 102–107), namely EBV-associated lymphoma (108). Additionally, HLA-II alleles associated with increased EBV susceptibility may also explain the higher prevalence of cancer and autoimmune disorders (**Figure 1C**), such as rheumatoid arthritis and type 1 diabetes, in first-degree relatives of patients with ME/CFS (15, 19, 100, 109, 110). Notwithstanding, the high prevalence of EBV among the general population hinders the identification of this putative subset of patients who developed ME/CFS following infection. This is in agreement with EBV serological assessments performed in patients and healthy individuals, from where no conclusive results have been observed (85). Similarly, most studies reported no increase in EBV viral load in patients with ME/CFS (84, 111). If this is due to the fact that the possible trigger of some EBV-associated diseases are the EBV-latent cells rather than viral load (34, 35, 43, 63, 66, 67, 112–114), remains unknown in ME/CFS. Thus, to our knowledge, there is scarce evidence based in prospective cohort studies, describing rates and associations with post-infective fatigue syndrome (i.e., ME/CFS) following proven acute EBV.

## Therapeutic Options Against EBV: Is There a Role in ME/CFS?

Limited knowledge about the origin of ME/CFS has hampered the development of effective treatment. Current strategies include administering nutritional supplements to overcome deficiencies and symptomatic treatment with analgesics, steroids or antidepressants (**Supplemental Table 3**) (1, 115). Thus, if a putative association between EBV infection and the onset of ME/CFS exists, the development of biomarkers that could identify patients in whom this may occur, would create a window of opportunity for tailored treatment against EBV.

B-cell depletion using several infusions of rituximab over 12 months was not associated with clinical improvement in patients with ME/CFS (116). Similarly, results of trials using antivirals have been inconclusive and, in some cases, contradictory (115). Accordingly, anecdotal observations of the resolution of symptoms in some ME/CFS patients with elevated levels of anti-EBV antibodies, after treatment with rituximab (100, 117) or antivirals (e.g., valaciclovir and valgancinclovir) (118, 119), have not been confirmed in large series. If B-cell depletion agents and antivirals were poised to show greater efficacy if used in a putative subgroup of patients in whom acute EBV triggered ME/CFS, remains obviously unknown. Furthermore there are no effective treatments to eradicate EBV latency in patients with EBV-associated disorders (120–122). Antiviral agents do not eradicate latent cells (120, 121), while rituximab does not remove all EBV-infected cells nor restores cellular immunity against EBV. Moreover, it induces further immunosuppression by targeting CD20 positive infected and uninfected healthy B cells (120, 122, 123). The combination of antiviral agents or intravenous immunoglobulins with rituximab can be used to treat EBV-associated disorders (121), but merely prolongs time until next relapse, since the combination of both therapies does not completely eliminate EBV infection in genetically predisposed hosts.

**Figure 2:**
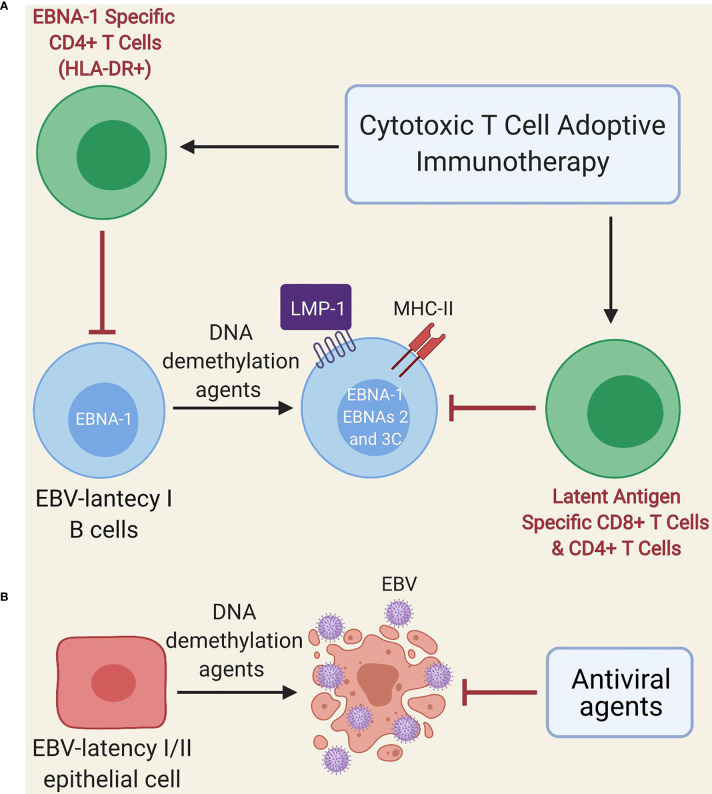
Schematic model of treatment with DNA demethylation agents followed by adoptive immunotherapy of EBV-specific T cells. **(A)** Administration of low-dose DNA demethylation agents (e.g. decitabine) restores the expression of MHC class II molecules and induces expression of LMP1, EBNA-2 and EBNA-3C in EBV-latency I B cells, improving the recognition of these cells by EBV-specific T cells (either autogenous or after adoptive immunotherapy). EBNA-1-specific CD4 T cells can only recognize latent I cells exhibiting EBNA-1 in MHC class II molecules since EBNA-1 is poorly immunogenic. **(B)** DNA demethylation agents (e.g. decitabine) induce lytic infection and apoptosis in EBV-transformed epithelial cells. Antiviral agents prevent viral replication.

Other treatments such as EBV-specific adoptive T cell immunotherapy could potentially yield some benefit (124), since EBNA-1 is expressed during the lytic cycle, as well as by almost all EBV-infected cells, with the exception of those with latency 0 (125–127). However, EBNA-1 is poorly immunogenic and is not always presented in MHC class II molecules of B cells with EBV latency (46); thus, these cells (especially those with latency I) could escape EBNA-1-specific T cell immunotherapy. Another alternative proposed by Dalton et al. is the use of low doses of DNA demethylation agents (e.g., decitabine and 5-azacitidine) during a short period (**Figure 2A**) for the treatment of latency I EBV-associated lymphoma, which induces the transformation of latent I B-cells into latent II and III, thereby allowing EBV-specific T cells to recognize them due to increased expression of immunogenic viral antigens (i.e., LMP1, EBNA2, EBNA3A and EBNA3C) (128). Such an effect could even persist after treatment interruption (128). Furthermore, DNA demethylation agents may restore the expression of HLA-II molecules in EBV-transformed lymphoblastoid cell lines (129, 130) and induce apoptosis in EBV-transformed epithelial cells (**Figure 2B**) (131, 132). Therefore, if our hypothesis about the presence of an acquired immunodeficiency after EBV infection in genetically predisposed individuals with EBV-associated diseases holds true, it could be speculated that low-dose DNA demethylation agents for short-term treatment followed by EBV-specific T cell immunotherapy (128) and antiviral agents (because of the increased lytic infection by the use of DNA demethylation agents) could be beneficial. Such an approach may also be potentially useful in cases where ME/CFS develops from other viruses such as the HHV-6, CMV and human parvovirus B19 (24–29), since these DNA viruses use CpG DNA methylation as an immune evasion mechanism. Namely, the viral genome is largely methylated during the latency phase thereby preventing viral expression and genome replication; instead, it is restored to a non-methylated state during the lytic phase during latency, which is restored to a non-methylated state during the lytic phase (133–136).

## Conclusions and Future Directions

If the link between EBV infection and ME/CFS could be demonstrated, it would warrant future research endeavors on a potential association between decreased activation of CD4 T cells and HLA class II alleles with greater predisposition to EBV infection. Such biomarkers could help to better define a hypothetical subgroup of patients with EBV-associated ME/CFS. Additional research about the efficacy of anti-EBV therapies in these patients would then be warranted. The identification of successful treatment would potentially prevent the development of this or other diseases associated with this pathogen. Interestingly, a recent prospective study investigating risk factors for developing ME/CFS in college students following infectious mononucleosis, found that those who developed ME/CFS had more physical symptoms and immune irregularities at baseline (137). If the association between the expression of certain HLA-II alleles and higher susceptibility to develop ME/CFS exists, individuals with post infectious mononucleosis fatigue syndrome could be an interesting group to study and test the hypothesis discussed in this review.

## Author Contributions

All the authors listed have made a substantial, direct, and intellectual contribution to this manuscript and have approved it for publication.

## Funding

Ramsay Award Program 2019 Cycle from the Solve ME/CFS Initiative.

## Conflict of Interest

The authors declare that the research was conducted in the absence of any commercial or financial relationships that could be construed as a potential conflict of interest.

## Publisher’s Note

All claims expressed in this article are solely those of the authors and do not necessarily represent those of their affiliated organizations, or those of the publisher, the editors and the reviewers. Any product that may be evaluated in this article, or claim that may be made by its manufacturer, is not guaranteed or endorsed by the publisher.
