# Supplementary material for: Epstein-Barr Virus and the Origin of Myalgic Encephalomyelitis or Chronic Fatigue Syndrome
Source: Front Immunol. 2021 Nov 15;12:656797. doi: 10.3389/fimmu.2021.656797 (PMC8634673; doi:10.3389/fimmu.2021.656797)
Supplement: Supplementary file 1 [file DataSheet_1.docx]

**Epstein-Barr virus and the origin of myalgic encephalomyelitis or chronic fatigue syndrome**

Manuel Ruiz-Pablos^1*^, Bruno Paiva^2^, Rosario Montero-Mateo^3^, Nicolas Garcia^2^, Aintzane Zabaleta^2*^

^1^European University of Madrid, Madrid, Spain

^2^Clinica Universidad de Navarra, Centro de Investigación Medica Aplicada (CIMA), IDISNA, Pamplona, Spain

^3^Hospital Clínico San Carlos, Madrid, Spain.

***Correspondence:**Manuel Ruiz Pablos: [manruipa@gmail.com](mailto:manruipa@gmail.com)

Aintzane Zabaleta: [azabaletaa@unav.es](mailto:azabaletaa@unav.es)

Centro de Investigación Médica Aplicada (CIMA)

Av. Pío XII 55, 31008 Pamplona, Spain

**Keywords:** chronic fatigue syndrome, myalgic encephalomyelitis, EBV EBNA-1, HLA, cancer, CD4+ CTL, autoimmunity, immunotherapy

**Supplemental Table 1.** Diagnostic criteria for Chronic Fatigue Syndrome or Myalgic Encephalomyelitis.

| Criteria | Main | Minor |
| --- | --- | --- |
| Holmes, 1988. (1) | ≥ Six months of severe chronic fatigue not due to exertion or other illness and which reduces or affects the activity of daily life by less than 50%. | ≥ Eight of the eleven minor symptoms:   - Fever or chills - Sore Throat - Tender lymph nodes - Muscle weakness - Muscle pain - Post-exertional malaise - Headaches - Migratory Arthralgia - Neuropsychiatric complaints - Sleep disturbances - Sudden onset of symptoms |
| Fukuda Criteria, 1994.(2) | ≥ Six months of severe chronic fatigue not due to exertion or other illness and which interferes with daily life | ≥ Four of the following eight symptoms:   - Sore throat - Tender cervical or axillary nodes - Muscle pain - Joint pain - Post-exertional malaise lasting more than 24 hours - Headaches - Impaired short-term memory or concentration - Unrefreshing sleep |
| Canadian Consensus Criteria for ME, 2003.(3) | ≥ Six months of:   - Persistent, unexplained, new-onset fatigue that reduces activity level, and/or - Post-exertional malaise, and/or - Sleep dysfunction and/or - Pain in the form of myalgia / generalized arthralgia or headaches | - ≥Two neurological/cognitive symptoms - Confusion - Impaired concentration and short-term memory - Difficulty in processing information, categorizing and retrieving words - Perceptive and sensory alterations - Ataxia - Muscle weakness - Fasciculations   and ≥ one of two of the following categories:   - Autonomic : orthostatic intolerance, PoTS, nausea, irritable bowel syndrome, urinary frequency, palpitations, stress dyspnea - Neuroendocrine: loss of thermostatic stability, weight change - Immunological: painful lymph nodes, recurrent sore throat, general malaise, food/drug sensitivity |
| International Consensus Criteria for ME (ME-ICC), 2011. (4) | Post-effort neuroimmune exhaustion with marked and prolonged post-effort physical or cognitive fatigue with a prolonged recovery period | ≥ A symptom of three of these categories:   - Neurocognitive impairment - Pain - Sleep disturbances - Neurosensory, perceptual and motor disorders   ≥ A symptom of three of these categories:   - Flu-like symptoms - Susceptibility to viral infections - Gastrointestinal Tract Symptoms - Genitourinary symptoms - Sensitivities   ≥ A symptom of these categories:   - Cardiovascular - Respiratory - Loss of thermostatic stability - Intolerance to extreme temperatures |

**Supplemental Table 2.** Main haplotypes related to genetic predisposition to develop diseases associated with EBV.

| **Haplotipes** | **EBV-associated diseases** |
| --- | --- |
| DR2-DQ6 (DRB1*1501, DQA1*0102, DQB1*0602). | It correlates positively with:   - Systemic lupus erythematosus (19–22). - Sjögren's syndrome (23,24). - Multiple Sclerosis (25,26). - Myalgic Encephalomyelitis/Chronic Fatigue Syndrome (27,28). - Late-onset/acquired myasthenia gravis (29,30). - Fulminant type diabetes (31). - Hodking lymphoma (32,33).   It correlates negatively with:   - Type 1 diabetes mellitus (31,34). - Graves' disease (35). |
| DR3-DQ2 (DRB1*0301, DQA1*0501, DQB1*0201). | It correlates positively with:   - Systemic lupus erythematosus (22). - Multiple sclerosis (36). - Diabetes mellitus type 1 (37,38). - Celiac disease (39–41). - Graves' disease (42,43). - Sjögren's Syndrome (23,24). - Early-onset myasthenia gravis (29,44,45). |
| DR4-DQ8 (DRB1*04, DQA1*03, DQB1*0302). | It correlates positively with   - Multiple Sclerosis (36). - Diabetes mellitus type 1 (37,38). - Celiac disease (39–41). - Rheumatoid arthritis (46,47) - Hashimoto's thyroiditis (42,43). |

**Supplemental Table 3.** Current therapeutic strategies for ME/CFS.

| **Medication** | **Examples** | **Effect** |
| --- | --- | --- |
| Corticosteroids | -Hydrocortisone  -Frudocortisone | Some improvement in ME/CFS symptoms, especially physical fatigue (5), but suppression of adrenal gland hormone production, can lead to depression and weight gain. |
| Supplements | -Nutritional supplements  -Acetyl-l-carnitine, -Essential fatty acids  -Magnesium  -Vitamins  -Coenzyme Q10 plus | Improvement of physical and mental fatigue in ME/CFS patients with specific nutritional deficiencies. (6,7) |
| NSAIDs | -Ibuprofen  -Naproxen  -Celecoxib | Relief of joint, muscle, and headache pain, reduces fever and inflammation (8). Chronic use can lead to ulcers, bleeding, and gastrointestinal symptoms. If ME/CFS had a viral origin, selective COX-2 inhibitors, such as celecoxib, could be useful in the treatment of latent infections, since COX-2 plays an important role in viral replication and is positively regulated during viral infection (9). |
| Antiviral Drugs | -Rintatolimod  -Valacyclovir  -Valganciclovir | Rintatolimod is a TLR3 agonist that improves exercise tolerance and allows a reduction in the concomitant use of CFS/ME related drugs by improving NK cell function and influencing the 2-5A-synthetase pathway (10,11).  ME/CFS patients with elevated antibodies to Epstein-Barr Virus (EBV) have experienced significant improvements in physical activity when taking valacyclovir (12).  Statistically significant improvements in cognitive function and a reduction in mental fatigue have been observed in ME/CFS patients who also had elevated serum IgG titers for EBV and human herpesvirus 6 (HHV-6) (13). |
| Monoclonal Antibodies | -Rituximab | Improvement of symptoms by decreasing activity and number of B cells (14,15). However, a subsequent Norwegian trial found no significant difference from placebo (16), which may be due to the inclusion criteria used, which currently do not differentiate the ME/CFS subgroups according to cause. It could benefit only the ME/CFS subgroup post EBV infection. Deletion of normal B cells may also increase the risk of other infections. |
| Selective serotonin-reuptake inhibitors | -Fluoxetine  -Sertraline  -Paroxetine | Used in those patients with ME/CFS who have anxiety, depression and other mood disorders, as well as patients with chronic neuropathic pain (17). They can generate insomnia, agitation and gastrointestinal symptoms. |
| Tricyclic antidepressants | -Amitriptyline  -Doxepin  -Nortriptyline  -Desipramine | It has an anxiolytic effect, improves locomotor activity, sleep, and relieves pain in lower doses than those used to treat depression (7,18). They can cause drowsiness, sedation, weight gain, urinary retention, cognitive impairment, and sexual dysfunction. |

**REFERENCES**

1. Holmes GP, Kaplan JE, Gantz NM, Komaroff AL, Schonberger LB, Straus SE, et al. Chronic fatigue syndrome: A working case definition. Ann Intern Med. 1988;108(3):387–9.

2. Fukuda K, Straus SE, Hickie I, Sharpe MC, Dobbins JG, Komaroff A. The chronic fatigue syndrome: A comprehensive approach to its definition and study. Ann Intern Med. 1994 Dec 15;121(12):953–9.

3. Carruthers BM, Jain AK, De Meirleir KL, Peterson DL, Klimas NG, Lemer AM, et al. Myalgic encephalomyelitis/chronic fatigue syndrome: Clinical working case definition, diagnostic and treatment protocols [Internet]. Vol. 11, Journal of Chronic Fatigue Syndrome. Taylor & Francis; 2003 [cited 2021 Jan 8]. p. 7–115. Available from: https://www.tandfonline.com/doi/abs/10.1300/J092v11n01_02

4. Carruthers BM, Van de Sande MI, De Meirleir KL, Klimas NG, Broderick G, Mitchell T, et al. Myalgic encephalomyelitis: International Consensus Criteria [Internet]. Vol. 270, Journal of Internal Medicine. Blackwell Publishing Ltd; 2011 [cited 2021 Jan 8]. p. 327–38. Available from: https://onlinelibrary.wiley.com/doi/full/10.1111/j.1365-2796.2011.02428.x

5. Chambers D, Bagnall AM, Hempel S, Forbes C. Interventions for the treatment, management and rehabilitation of patients with chronic fatigue syndrome/myalgic encephalomyelitis: An updated systematic review [Internet]. Vol. 99, Journal of the Royal Society of Medicine. J R Soc Med; 2006 [cited 2021 Jan 8]. p. 506–20. Available from: https://pubmed.ncbi.nlm.nih.gov/17021301/

6. Beth Smith ME, Haney E, McDonagh M, Pappas M, Daeges M, Wasson N, et al. Treatment of myalgic encephalomyelitis/chronic fatigue syndrome: A systematic review for a National Institutes of health pathways to prevention workshop [Internet]. Vol. 162, Annals of Internal Medicine. American College of Physicians; 2015 [cited 2021 Jan 8]. p. 841–50. Available from: http://annals.org/article.aspx?doi=10.7326/M15-0114

7. Cortes Rivera M, Mastronardi C, Silva-Aldana C, Arcos-Burgos M, Lidbury B. Myalgic Encephalomyelitis/Chronic Fatigue Syndrome: A Comprehensive Review. Diagnostics [Internet]. 2019 Aug 7 [cited 2021 Jan 8];9(3):91. Available from: https://www.mdpi.com/2075-4418/9/3/91

8. Theoharides TC, Asadi S, Weng Z, Zhang B. Serotonin-Selective Reuptake Inhibitors and Nonsteroidal Anti-Inflammatory Drugs-Important Considerations of Adverse Interactions Especially for the Treatment of Myalgic Encephalomyelitis/Chronic Fatigue Syndrome. J Clin Psychopharmacol [Internet]. 2011 Aug [cited 2021 Jan 8];31(4):403–5. Available from: http://journals.lww.com/00004714-201108000-00001

9. Richman S, Morris MC, Broderick G, Craddock TJA, Klimas NG, Fletcher MA. Pharmaceutical Interventions in Chronic Fatigue Syndrome: A Literature-based Commentary. Clin Ther [Internet]. 2019 May 1 [cited 2021 Jan 8];41(5):798–805. Available from: https://doi.org/10.1016/j.clinthera.2019.02.011

10. Strayer DR, Carter WA, Stouch BC, Stevens SR, Bateman L, Cimoch PJ, et al. A double-blind, placebo-controlled, randomized, clinical trial of the TLR-3 agonist rintatolimod in severe cases of chronic fatigue syndrome. PLoS One [Internet]. 2012 Mar 14 [cited 2021 Jan 8];7(3). Available from: https://pubmed.ncbi.nlm.nih.gov/22431963/

11. Mitchell WM. Efficacy of rintatolimod in the treatment of chronic fatigue syndrome/myalgic encephalomyelitis (CFS/ME). Expert Rev Clin Pharmacol [Internet]. 2016 Jun 2 [cited 2021 Jan 8];9(6):755–70. Available from: https://www.tandfonline.com/doi/full/10.1586/17512433.2016.1172960

12. Lerner AM, Beqaj SH, Deeter RG, Fitzgerald JT. Valacyclovir treatment in Epstein-Barr virus subset chronic fatigue syndrome: Thirty-six months follow-up. In Vivo (Brooklyn) [Internet]. 2007 Sep 1;21(5):707–14. Available from: https://europepmc.org/article/med/18019402

13. Montoya JG, Kogelnik AM, Bhangoo M, Lunn MR, Flamand L, Merrihew LE, et al. Randomized clinical trial to evaluate the efficacy and safety of valganciclovir in a subset of patients with chronic fatigue syndrome. J Med Virol [Internet]. 2013 Dec 1 [cited 2021 Jan 8];85(12):2101–9. Available from: http://doi.wiley.com/10.1002/jmv.23713

14. Fluge Ø, Bruland O, Risa K, Storstein A, Kristoffersen EK, Sapkota D, et al. Benefit from B-Lymphocyte Depletion Using the Anti-CD20 Antibody Rituximab in Chronic Fatigue Syndrome. A Double-Blind and Placebo-Controlled Study. Reindl M, editor. PLoS One [Internet]. 2011 Oct 19 [cited 2021 Jan 8];6(10):e26358. Available from: https://dx.plos.org/10.1371/journal.pone.0026358

15. Fluge Ø, Risa K, Lunde S, Alme K, Rekeland IG, Sapkota D, et al. B-Lymphocyte Depletion in Myalgic Encephalopathy/ Chronic Fatigue Syndrome. An Open-Label Phase II Study with Rituximab Maintenance Treatment. van der Feltz-Cornelis C, editor. PLoS One [Internet]. 2015 Jul 1 [cited 2021 Jan 8];10(7):e0129898. Available from: https://dx.plos.org/10.1371/journal.pone.0129898

16. Fluge Ø, Rekeland IG, Lien K, Thürmer H, Borchgrevink PC, Schäfer C, et al. B-lymphocyte depletion in patients with myalgic encephalomyelitis/chronic fatigue syndrome: A randomized, double-blind, placebo-controlled trial. Ann Intern Med. 2019;170(9):585–93.

17. Li DQ, Li ZC, Dai ZY. Selective serotonin reuptake inhibitor combined with dengzhanshengmai capsule improves the fatigue symptoms: A 12-week open-label pilot study. Int J Clin Exp Med [Internet]. 2015;8(7):11811–7. Available from: www.ijcem.com/

18. Kumar A, Garg R. Protective effects of antidepressants against chronic fatigue syndrome - induced behavioral changes and biochemical alterations. Fundam Clin Pharmacol [Internet]. 2009 Feb 1 [cited 2021 Jan 8];23(1):89–95. Available from: http://doi.wiley.com/10.1111/j.1472-8206.2008.00638.x

19. Al-Motwee S, Jawdat D, Jehani GS, Anazi H, Shubaili A, Sutton P, et al. Association of HLA-DRB1*15 and HLA-DQB1* 06 with SLE in Saudis. Ann Saudi Med [Internet]. 2013 May [cited 2021 Jan 8];33(3):229–34. Available from: http://www.annsaudimed.net/doi/10.5144/0256-4947.2013.229

20. Liphaus BL, Kiss MHB, Goldberg AC. HLA-DRB1 alleles in juvenile-onset systemic lupus erythematosus: Renal histologic class correlations. Brazilian J Med Biol Res [Internet]. 2007 [cited 2021 Jan 8];40(4):591–7. Available from: www.bjournal.com.br

21. Shimane K, Kochi Y, Suzuki A, Okada Y, Ishii T, Horita T, et al. An association analysis of HLA-DRB1 with systemic lupus erythematosus and rheumatoid arthritis in a Japanese population: effects of *09:01 allele on disease phenotypes. Rheumatology [Internet]. 2013 Jul 1 [cited 2021 Jan 8];52(7):1172–82. Available from: https://academic.oup.com/rheumatology/article-lookup/doi/10.1093/rheumatology/kes427

22. Morris DL, Taylor KE, Fernando MMA, Nititham J, Alarcón-Riquelme ME, Barcellos LF, et al. Unraveling multiple MHC gene associations with systemic lupus erythematosus: Model choice indicates a role for HLA alleles and non-HLA genes in europeans. Am J Hum Genet [Internet]. 2012 Nov 2 [cited 2021 Jan 8];91(5):778–93. Available from: http://dx.doi.org/10.1016/j.ajhg.2012.08.026.

23. Cobb BL, Lessard CJ, Harley JB, Moser KL. Genes and Sjögren’s Syndrome [Internet]. Vol. 34, Rheumatic Disease Clinics of North America. Rheum Dis Clin North Am; 2008 [cited 2021 Jan 8]. p. 847–68. Available from: https://pubmed.ncbi.nlm.nih.gov/18984408/

24. Guggenbuhl P, Jean S, Jego P, Grosbois B, Chalès G, Semana G, et al. Primary Sjogren’s syndrome: Role of the HLA-DRB1 0301-1501 heterozygotes. J Rheumatol [Internet]. 1998 May 1;25(5):900–5. Available from: https://europepmc.org/article/med/9598888

25. Hollenbach JA, Oksenberg JR. The immunogenetics of multiple sclerosis: A comprehensive review. Vol. 64, Journal of Autoimmunity. Academic Press; 2015. p. 13–25.

26. Lünemann JD, Jelčić I, Roberts S, Lutterotti A, Tackenberg B, Martin R, et al. EBNA1-specific T cells from patients with multiple sclerosis cross react with myelin antigens and co-produce IFN-γ and IL-2. J Exp Med [Internet]. 2008 Aug 4 [cited 2021 Jan 8];205(8):1763–73. Available from: www.jem.org/cgi/doi/10.1084/jem.20072397

27. Smith J, Fritz EL, Kerr JR, Cleare AJ, Wessely S, Mattey DL. Association of chronic fatigue syndrome with human leucocyte antigen class II alleles. J Clin Pathol [Internet]. 2005 Aug 1 [cited 2021 Jan 8];58(8):860–3. Available from: www.jclinpath.com

28. Underhill JA, Mahalingam M, Peakman M, Wessely S. Lack of association between HLA genotype and chronic fatigue syndrome. Eur J Immunogenet [Internet]. 2001 Jun 1 [cited 2021 Jan 8];28(3):425–8. Available from: http://doi.wiley.com/10.1046/j.1365-2370.2001.00235.x

29. Maniaol AH, Elsais A, Lorentzen ÅR, Owe JF, Viken MK, Sæther H, et al. Late Onset Myasthenia Gravis Is Associated with HLA DRB1*15:01 in the Norwegian Population. Tang J, editor. PLoS One [Internet]. 2012 May 9 [cited 2021 Jan 8];7(5):e36603. Available from: https://dx.plos.org/10.1371/journal.pone.0036603

30. Misra MK, Damotte V, Hollenbach JA. The immunogenetics of neurological disease. Immunology [Internet]. 2018 Apr 1 [cited 2021 Jan 8];153(4):399–414. Available from: http://doi.wiley.com/10.1111/imm.12869

31. Fujiya A, Ochiai H, Mizukoshi T, Kiyota A, Shibata T, Suzuki A, et al. Fulminant type 1 diabetes mellitus associated with a reactivation of Epstein-Barr virus that developed in the course of chemotherapy of multiple myeloma. J Diabetes Investig [Internet]. 2010 Dec 1 [cited 2021 Jan 8];1(6):286–9. Available from: http://doi.wiley.com/10.1111/j.2040-1124.2010.00061.x

32. Johnson PCD, McAulay KA, Montgomery D, Lake A, Shield L, Gallagher A, et al. Modeling HLA associations with EBV‐positive and ‐negative <scp>H</scp> odgkin lymphoma suggests distinct mechanisms in disease pathogenesis. Int J Cancer [Internet]. 2015 Sep 20 [cited 2021 Jan 8];137(5):1066–75. Available from: https://onlinelibrary.wiley.com/doi/abs/10.1002/ijc.29467

33. McAulay KA, Jarrett RF. Human leukocyte antigens and genetic susceptibility to lymphoma. Tissue Antigens [Internet]. 2015 Aug 1 [cited 2021 Jan 8];86(2):98–113. Available from: http://doi.wiley.com/10.1111/tan.12604

34. Noble JA, Valdes AM. Genetics of the HLA region in the prediction of type 1 diabetes. Curr Diab Rep [Internet]. 2011 Dec 13 [cited 2021 Jan 8];11(6):533–42. Available from: https://link.springer.com/article/10.1007/s11892-011-0223-x

35. Badenhoop K, Walfish PG, Rau H, Fischer S, Nicolay A, Bogner U, et al. Susceptibility and resistance alleles of human leukocyte antigen (HLA) DQA1 and HLA DQB1 are shared in endocrine autoimmune disease. J Clin Endocrinol Metab [Internet]. 1995 Jul 1 [cited 2021 Jan 8];80(7):2112–7. Available from: https://academic.oup.com/jcem/article-lookup/doi/10.1210/jcem.80.7.7608264

36. Luckey D, Bastakoty D, Mangalam AK. Role of HLA class II genes in susceptibility and resistance to multiple sclerosis: Studies using HLA transgenic mice. J Autoimmun. 2011 Sep 1;37(2):122–8.

37. Pociot F, Lernmark Å. Genetic risk factors for type 1 diabetes [Internet]. Vol. 387, The Lancet. Lancet Publishing Group; 2016 [cited 2021 Jan 8]. p. 2331–9. Available from: http://www.thelancet.com/article/S0140673616305827/fulltext

38. Burrack AL, Martinov T, Fife BT. T cell-mediated beta cell destruction: Autoimmunity and alloimmunity in the context of type 1 diabetes [Internet]. Vol. 8, Frontiers in Endocrinology. Frontiers Media S.A.; 2017 [cited 2021 Jan 8]. p. 1. Available from: www.frontiersin.org

39. Camarca ME, Mozzillo E, Nugnes R, Zito E, Falco M, Fattorusso V, et al. Celiac disease in type 1 diabetes mellitus. [Internet]. Vol. 38, Italian journal of pediatrics. BioMed Central; 2012 [cited 2021 Jan 8]. p. 10. Available from: http://ijponline.biomedcentral.com/articles/10.1186/1824-7288-38-10

40. Smigoc Schweiger D, Mendez A, Kunilo Jamnik S, Bratanic N, Bratina N, Battelino T, et al. High-risk genotypes HLA-DR3-DQ2/DR3-DQ2 and DR3-DQ2/DR4-DQ8 in co-occurrence of type 1 diabetes and celiac disease. Autoimmunity [Internet]. 2016 May 18 [cited 2021 Jan 8];49(4):240–7. Available from: https://www.tandfonline.com/doi/abs/10.3109/08916934.2016.1164144

41. Liu E, Lee H-S, Aronsson CA, Hagopian WA, Koletzko S, Rewers MJ, et al. Risk of Pediatric Celiac Disease According to HLA Haplotype and Country. N Engl J Med [Internet]. 2014 Jul 3 [cited 2021 Jan 8];371(1):42–9. Available from: https://www.nejm.org/doi/10.1056/NEJMoa1313977

42. Zeitlin AA, Heward JM, Newby PR, Carr-Smith JD, Franklyn JA, Gough SCL, et al. Analysis of HLA class II genes in Hashimoto’s thyroiditis reveals differences compared to Graves’ disease. Genes Immun [Internet]. 2008 Jun 1 [cited 2021 Jan 8];9(4):358–63. Available from: www.nature.com/gene

43. El-Ahwal L, AbdEL-Bar E. The frequency of HLA DRB1-DQB1 alleles in autoimmune type 1 diabetes with or without autoimmune thyroid disease. Tanta Med J [Internet]. 2015 [cited 2021 Jan 8];43(2):66. Available from: http://www.tdj.eg.net/article.asp?issn=1110-1415;year=2015;volume=43;issue=2;spage=66;epage=71;aulast=El-Ahwal

44. Zagoriti Z, Kambouris ME, Patrinos GP, Tzartos SJ, Poulas K. Recent advances in genetic predisposition of myasthenia gravis. Vol. 2013, BioMed Research International. 2013. p. 404053.

45. Hjelmström P, Giscombe R, Lefvert AK, Pirskanen R, Kockum I, Landin-Olsson M, et al. Different HLA-DQ are positively and negatively associated in swedish patients with myasthenia gravis. Autoimmunity [Internet]. 1995 [cited 2021 Jan 8];22(1):59–65. Available from: https://www.tandfonline.com/doi/abs/10.3109/08916939508995300

46. Chemin K, Gerstner C, Malmström V. Effector functions of CD4+ T cells at the site of local autoimmune inflammation-lessons from rheumatoid arthritis. Front Immunol [Internet]. 2019 Mar 12 [cited 2021 Jan 8];10:353. Available from: www.frontiersin.org

47. Trier N, Izarzugaza J, Chailyan A, Marcatili P, Houen G. Human MHC-II with Shared Epitope Motifs Are Optimal Epstein-Barr Virus Glycoprotein 42 Ligands—Relation to Rheumatoid Arthritis. Int J Mol Sci [Internet]. 2018 Jan 21 [cited 2021 Jan 8];19(1):317. Available from: http://www.mdpi.com/1422-0067/19/1/317
